# Dysbiosis and structural disruption of the respiratory microbiota in COVID-19 patients with severe and fatal outcomes

**DOI:** 10.1038/s41598-021-00851-0

**Published:** 2021-10-29

**Authors:** Alejandra Hernández-Terán, Fidencio Mejía-Nepomuceno, María Teresa Herrera, Omar Barreto, Emma García, Manuel Castillejos, Celia Boukadida, Margarita Matias-Florentino, Alma Rincón-Rubio, Santiago Avila-Rios, Mario Mújica-Sánchez, Ricardo Serna-Muñoz, Eduardo Becerril-Vargas, Cristobal Guadarrama-Pérez, Víctor Hugo Ahumada-Topete, Sebastián Rodríguez-Llamazares, José Arturo Martínez-Orozco, Jorge Salas-Hernández, Rogelio Pérez-Padilla, Joel Armando Vázquez-Pérez

**Affiliations:** 1 Departamento de Investigación en Tabaquismo y EPOC, Instituto Nacional de Enfermedades Respiratorias Ismael Cosío Villegas, INER, Mexico, Mexico; 2 Departamento de Investigación en Microbiología, Instituto Nacional de Enfermedades Respiratorias Ismael Cosío Villegas, INER, Mexico, Mexico; 3 Coordinación de Atención Médica, Instituto Nacional de Enfermedades Respiratorias Ismael Cosío Villegas, INER, Mexico, Mexico; 4 Departamento de Unidad de Epidemiología Hospitalaria e Infectología, Instituto Nacional de Enfermedades Respiratorias Ismael Cosío Villegas, INER, Mexico, Mexico; 5 Centro de Investigación en Enfermedades Infecciosas, CIENI, Instituto Nacional de Enfermedades Respiratorias Ismael Cosío Villegas, INER, Mexico, Mexico; 6 Laboratorio de Microbiología, Instituto Nacional de Enfermedades Respiratorias Ismael Cosío Villegas, INER, Mexico, Mexico; 7 Servicio de Urgencias Médicas, Instituto Nacional de Enfermedades Respiratorias Ismael Cosío Villegas, INER, Mexico, Mexico; 8 Dirección General INER, Instituto Nacional de Enfermedades Respiratorias Ismael Cosío Villegas, INER, Mexico, Mexico

**Keywords:** Clinical microbiology, Viral infection

## Abstract

The COVID-19 outbreak has caused over three million deaths worldwide. Understanding the pathology of the disease and the factors that drive severe and fatal clinical outcomes is of special relevance. Studying the role of the respiratory microbiota in COVID-19 is especially important as the respiratory microbiota is known to interact with the host immune system, contributing to clinical outcomes in chronic and acute respiratory diseases. Here, we characterized the microbiota in the respiratory tract of patients with mild, severe, or fatal COVID-19, and compared it to healthy controls and patients with non-COVID-19-pneumonia. We comparatively studied the microbial composition, diversity, and microbiota structure between the study groups and correlated the results with clinical data. We found differences in the microbial composition for COVID-19 patients, healthy controls, and non-COVID-19 pneumonia controls. In particular, we detected a high number of potentially opportunistic pathogens associated with severe and fatal levels of the disease. Also, we found higher levels of dysbiosis in the respiratory microbiota of patients with COVID-19 compared to the healthy controls. In addition, we detected differences in diversity structure between the microbiota of patients with mild, severe, and fatal COVID-19, as well as the presence of specific bacteria that correlated with clinical variables associated with increased risk of mortality. In summary, our results demonstrate that increased dysbiosis of the respiratory tract microbiota in patients with COVID-19 along with a continuous loss of microbial complexity structure found in mild to fatal COVID-19 cases may potentially alter clinical outcomes in patients. Taken together, our findings identify the respiratory microbiota as a factor potentially associated with the severity of COVID-19.

## Introduction

The Coronavirus Disease 2019 (COVID-19) outbreak, declared a pandemic by the World Health Organization on March 11, 2020, is caused by the Severe Acute Respiratory Syndrome Coronavirus 2 (SARS-CoV-2). As of May 2021, SARS-CoV-2 has infected more than 150 million people and caused over three million deaths worldwide^1^. COVID-19 shows a broad spectrum of clinical manifestations ranging from asymptomatic infection and mild respiratory symptoms to severe pneumonia and death^2,3^ and has been linked to demographic factors and comorbidities^4,5^. To date, aberrant immune responses against SARS-CoV-2 antigens have been shown to be critically involved in severe clinical outcomes and other secondary inflammatory conditions that remain after initial COVID-19 infection^3,6^.

Studying the role of the human microbiota in COVID-19 is particularly relevant, as the respiratory microbiota is known to interact with the host immune system, contributing to clinical outcomes in chronic and acute respiratory diseases^7^. The respiratory microbiota plays a central role in shaping pulmonary immunity by enhancing innate and adaptive immune responses. This suggests that host immunity is regulated by interactions with bacterial communities in the respiratory tract.

Some studies suggest that interactions between microorganisms and the host immune system are species-specific, denoting that even small variations in the diversity and composition of the microbiota could have important consequences on host health^7–9^. In the case of COVID-19, severe to fatal clinical outcomes are often associated with the presence of comorbidities known to exhibit an altered (dysbiotic) microbiota^10^ (e.g., diabetes type II, obesity, age, and heart disease). Furthermore, in a wide range of microbiome-associate diseases (MADs), dysbiosis is a common feature that may impact disease progression^11,12^. Nonetheless, few studies that characterize the respiratory microbiota in COVID-19 and the presence of dysbiosis are available to date^13–20^.

To gain insight into the association between respiratory microbiota and COVID-19 severity, we characterized the microbiota in the respiratory tract of patients with mild, severe, or fatal COVID-19, and compared it with healthy controls and patients with non-COVID-19-pneumonia. We performed comprehensive analyses to evaluate the respiratory microbiota as a risk factor and its implications for patients with COVID-19. We comparatively studied the microbial composition, diversity, and structure of the microbiota between the study groups and correlated the results with clinical data. These analyses let us detect firstly, differences in bacterial abundance between groups, secondly, higher levels of dysbiosis in the respiratory microbiota of patients with COVID-19, thirdly, differences in diversity structure between the microbiota of patients with mild, severe, and fatal COVID-19, and lastly, the presence of specific bacteria that correlated with clinical variables associated with increased risk of mortality. In summary, our results demonstrate that increased dysbiosis of the respiratory tract microbiota in patients with COVID-19, along with a continuous loss of microbial complexity structure found in mild to fatal COVID-19 cases, may potentially alter clinical outcomes in patients. Taken together, our findings identify the respiratory microbiota as a factor potentially associated with the severity of COVID-19.

## Results

### Study participants

Since our sample set consists of upper and lower respiratory tract samples, we retained only upper respiratory samples for main diversity and statistical analyses. In total, 95 samples were analyzed (mild COVID-19 = 37, severe COVID-19 = 27, fatal COVID-19 = 19, healthy controls = 7, and non-COVID-19-pneumonia = 5).

Demographics, health-related characteristics, and symptomatology are described in Table 1. Overall, 52 patients were male (54.7%) with a median age of 45 years old (IQR: 21). Regarding health conditions, 58.2% of the participants had at least one comorbidity, with DM2 (17%), hypertension (17%), smoking (17%), and obesity (35%) being the most represented in the cohort. The median days of symptom onset was seven, and 52.6% of the individuals received antibiotic treatment prior to hospitalization. In addition, we found important associations between some health/demographic characteristics and severity. For example, patients with fatal COVID-19 were predominantly male (73.6%, Wilcoxon rank-sum test *p* = 0.01), significantly older (median = 58, Wilcoxon rank-sum test *p* = 6.57e−07), with higher BMI (median = 30.4, Wilcoxon rank-sum test *p* = 0.05), and most of them received prior antibiotic treatment (78.9%, Wilcoxon rank-sum test *p* = 0.002) compared to patients with severe and mild COVID-19. Also, a higher number of days after symptoms onset was found in the non-COVID-19 pneumonia group (median = 10, Wilcoxon rank-sum test *p* = 0.01).

**Table 1:** Demographic data of the cohort.

	All (N = 95)	Healthy control (N = 7)	COVID-19 Mild (N = 37)	COVID-19 Severe (N = 27)	COVID-19 Fatal (N = 19)	Non-COVID-19-pneumonia (N = 5)	*p* value
**Age (years), median (IQR)**	45 (21)	35 (18)	37 (18)	47 (21)	58 (16.5)	49 (13)	6.75e−07***
**Gender**							
Female, n (%)	43 (45.2%)	5 (71.4%)	18 (48.6%)	12 (44%)	5 (26.3%)	3 (60%)	0.016*
Male, n (%)	52 (54.7%)	2 (28.5%)	19 (51.3%)	15 (55.5%)	14 (73.6%)	2 (40%)	
**Smoking, n (%)**	15 (17%)	0	0	8 (29.6%)	7 (36.8%)	0	ns
NA	8	1	4	0	1	2	
**BMI (kg/m**^**2**^**), median (IQR)**	27.6 (6.9)	26.2 (2.6)	27.34 (7.9)	28.9 (4.6)	30.4 (9.8)	24.9 (2.3)	0.05*
**Obesity**							
Not obese, n(%)	48 (50.5%)	5 (71.4%)	16 (43.2%)	16 (59.2%)	8 (42.1%)	3 (60%)	ns
Class I, n (%)	16 (61.5%)	0	9 (81.8%)	7 (77.7%)	2 (25%)	0	
Class II, n(%)	7 (29.9%)	0	1 (9%)	2 (22%)	4 (50%)	0	
Class III, n(%)	3 (11.5%)	0	1 (9%)	0	2 (25%)	0	
NA	21	2	12	2	3	2	
**Comorbidities**							
DM2, n (%)	15 (17%)	0	3 (8%)	7 (70.3%)	5 (26.3%)	0	ns
Hypertension, n (%)	15 (17%)	0	3 (8%)	4 (14.8%)	8 (42.1%)	0	ns
Respiratory disease*, n (%)	4 (4.5%)	0	3 (8%)	1 (3.7%)	1 (5.2%)	0	ns
NA	36	2	17	1	8	8	
**Days after symptoms onset, n (IQR)**	7 (6)	NA	5 (5)	6.5 (4.7)	5 (5)	10 (3)	0.01*
**Antibiotic treatment, n (%)**	50 (52.6%)	1 (14.2%)	11 (29.7%)	21 (77.7%)	15 (78.9%)	2 (40%)	0.002**
**Symptoms**							
Cough, n (%)	50 (52%)	2 (28.5%)	11 (29.7%)	20 (74%)	15 (78.9%)	2 (40%)	ns
Fever, n (%)	48 (50.5%)	2 (28.5%)	10 (27%)	19 (70.3%)	15 (78.9%)	2 (40%)	ns
Dyspnea, n (%)	42 (44%)	0	4 (10.8%)	18 (66.6%)	17 (89%)	3 (60%)	ns
Headache, n (%)	40 (42%)	1 (14.2%)	15 (40.5%)	13 (48%)	11 (57.8%)	0	0.001**
Myalgia, n (%)	38 (40%)	2 (28.5%)	12 (32.4%)	13 (48%)	10 (52.6%)	1 (20%)	0.003**
Arthralgia, n (%)	36 (37.8%)	2 (28.5%)	10 (27%)	13 (48%)	10 (52.6%)	1 (20%)	0.04*
Fatigue, n (%)	20 (21%)	0	3 (8%)	8 (29.6%)	9 (47%)	0	ns
Rhinorrhea, n (%)	26 (27.3%)	1 (14.2%)	6 (16%)	12 (44%)	6 (31.5%)	1 (20%)	ns
Chest pain, n (%)	13 (13.6%)	0	5 (13%)	4 (14.8%)	4 (21%)	0	ns
Diarrhea, n (%)	16 (16.8%)	0	4 (10.8%)	8 (29.6%)	4 (21%)	0	ns
Cyanosis, n (%)	7 (7.3%)	0	0	3(11%)	4 (21%)	0	ns
Vomiting, n (%)	5 (5.2%)	0	1 (2.7%)	3 (11%)	1 (5.2%)	0	ns
NA	105	0	70	12	0	23	

Only upper respiratory samples (OPS and NPS) are included in the information. *BMI* Body Mass Index, *DM2* Diabetes Mellitus Type 2. *Respiratory diseases: either asthma, COPD, or ILD. *P* values denote statistical significant differences given by Wilcoxon rank-sum test (*< 0.05, **< 0.005, ***< 0.0005).

### Respiratory microbiota composition differs between severity levels of COVID-19 patients and controls

**Figure 1:**
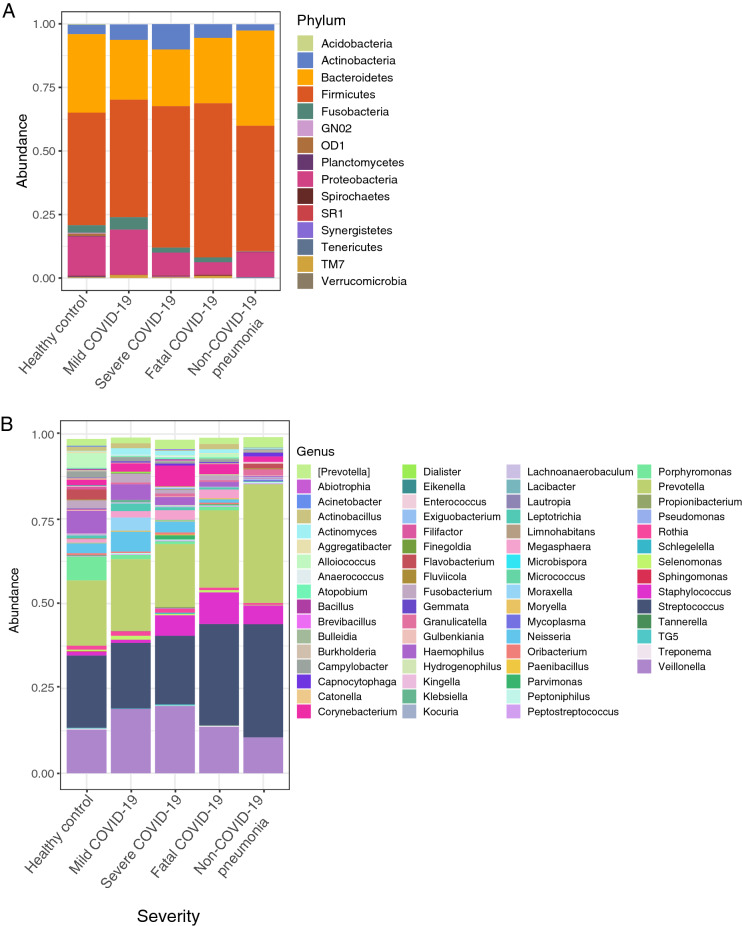
Microbial composition at phylum and genus level between patients with different severity levels of COVID-19 and controls. (**A**) Stacked barplot summarizing microbial composition between groups at phylum level. (**B**) Stacked barplot summarizing microbial composition between groups at genus level. Each color represents a taxa described in Phylum and Genus legends.

Of the 95 analyzed samples belonging to the upper respiratory tract, we identified a total of 4514 Amplicon Sequence Variants (ASVs). Regarding the analysis of the relative abundance at the phylum level (Fig. 1A), Firmicutes, Bacteroidetes, and Proteobacteria were the most dominant phyla among our severity groups and controls. In general, these phyla are present in all group samples but there were changes in relative abundance associated with disease severity. Overall, we found Firmicutes, Actinobacteria, and TM7 to be increased in patients with COVID-19, while Bacteroidetes and Proteobacteria were found to be decreased.

Analysis of relative abundance at the genus level revealed genera that differed significantly between patients with COVID-19 patients and controls (Fig. 1B). Overall, we found *Veillonella*, *Staphylococcus, Corynebacterium, Neisseria*, *Actinobacillu*s, and *Selenomonas* enriched in patients with COVID-19 but reduced in the healthy controls. Conversely, we found *Haemophilus* and *Alloiococcus* enriched in the healthy controls but reduced in patients with COVID-19. In addition, there were differences in the abundance of some genera between the severity levels for COVID-19. For example, *Streptococcus* and *Staphylococcus* showed increasing abundance from mild to fatal COVID-19. By contrast, *Haemophilus* and *Actinomyces* showed the opposite pattern, where the highest abundance was associated with mild COVID-19 and the lowest with fatal COVID-19. In addition, we found *Corynebacterium* highly abundant only in severe COVID-19, whereas *Actinobacillus* was found highly abundant only in fatal and mild COVID-19.

Additionally, we also characterized the lower respiratory tract microbiota between patients with severe and fatal COVID-19, finding a different composition from that found in the upper respiratory tract (Supplementary Fig. S1). We found that fatal patients showed a higher abundance of Proteobacteria and Bacteroidetes compared to severe patients (Supplementary Fig. S1A), contrary to our findings in the URT characterization. At the genus level, we found that fatal patients showed a higher abundance of *Prevotella, Staphylococcus, Haemophilus*, and *Enterococcus*, while severe patients showed a higher abundance of *Streptococcus* and *Abiotrophia.* Regarding the LefSe analysis, we found significant decreases of *Veillonella parvula* and *V. dispar* in fatal patients, while in severe patients we found members of the genera *Streptococcus, Neisseria, Abiotrophia,* and *Actinobacillus* significantly increased. However, it is worth mentioning that regardless of the differences found in phyla and genus abundances, we found no differences in alpha and beta diversity analyses (Supplementary Fig. S1C,E).

#### Alpha diversity

**Figure 2:**
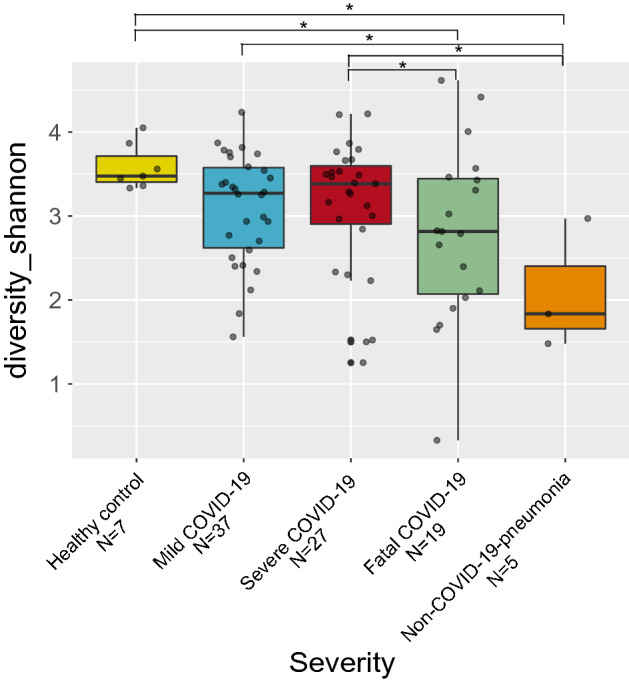
Alpha diversity of the respiratory microbiota between patients with different severity levels of COVID-19 and controls. Boxplot of the Shannon–Wiener index value for all analyzed groups. Asterisks denote statistical significant differences given by Wilcoxon rank-sum test (*p < 0.05).

Concerning diversity calculated with the Shannon–Wiener index (Fig. 2), we found healthy controls as the most diverse group and the non-COVID-19 pneumonia group (Wilcoxon rank-sum test, *p* < 0.05) as the least diverse. Although between severity groups the differences are not considerable, we found significant differences between the severe and fatal COVID-19 groups (Wilcoxon rank-sum test, *p* < 0.05).

#### Beta diversity

**Figure 3:**
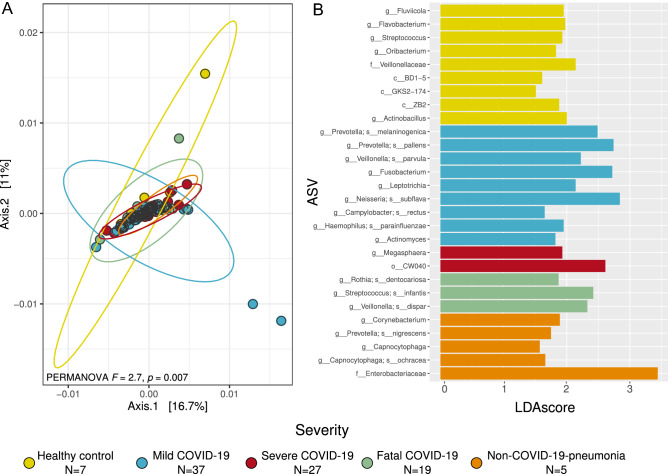
Beta diversity of the respiratory microbiota between patients with different severity levels of COVID-19 and controls. (**A**) Principal Coordinates Analysis (PCoA) with weighted Unifrac distance and PERMANOVA result that test differences in the community arrange between groups. Each color represents an analyzed group specified in the legend. (**B**) Differentially abundant taxa for each group obtained through LefSe analysis. Only features with a LDA score higher than 1.5 and a *p* < 0.01 were included. *ASV* Amplicon Sequence Variant.

Beta diversity analyses showed differences in the microbiota composition between patients with different severity levels of COVID-19 and controls (Fig. 3A,B, Supplementary Fig. S2). In particular, the PCoA analysis (Fig. 3A) showed differences between severity levels and control groups which are supported by the PERMANOVA result (F = 2.7, *p* = 0.007). We observe changes in the direction of the ellipses belonging to mild COVID-19 and healthy controls. While the ellipses for all severe, fatal COVID-19 and non-COVID-19 pneumonia occurred in the same direction, the ellipse for mild COVID-19 was almost orthogonal. Furthermore, when analyzing the results of the Ružička metric we detected that the intra-treatment similarities of the healthy controls were significantly higher than the similarities between the diseased samples, meaning that the COVID-19 associated microbiota (regardless of severity level) exhibited significantly higher levels of dysbiosis compared to the healthy controls (Supplementary Fig. S2).

The LefSe analysis allowed us to identify the differentially abundant taxa associated with the groups that were compared (Fig. 3B). We observed that all the severe COVID-19 groups and the two control groups showed differentially abundant taxa or biomarkers. In particular, for mild COVID-19, we found *Prevotella melaninogenica* and *P. pallens*, *Veillonella parvula*, *Neisseria subflava*, *Fusobacterium*, and* Actinomyces* to be highly abundant. In the case of severe COVID-19, we found *Megasphaera* and CW040 as the most prevalent. In the case of fatal COVID-19, *Rothia dentocariosa, Streptococcus infantis*, and *Veillonella dispar* were the most significant. In addition, the highest number of differentially abundant taxa was found to be associated with healthy controls (e.g., *Streptococcus*, *Flavobacterium*, and *Oribacterium*, and f_*Veillonellaceae*). Finally, for the non-COVID-19-pneumonia group, we found *Corynebacterium*, *Prevotella nigrescens*, *Capnocytophaga*, and *Enterobacteriaceae* to be the most abundant.

### Clinical variables associated with mortality risk correlated with specific microbial groups of the respiratory microbiota

**Figure 4:**
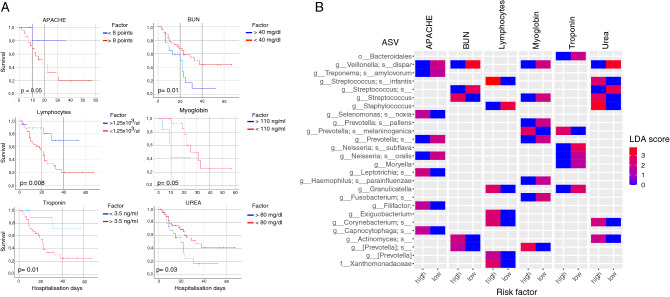
Correlation between clinical variables affecting probability of survival and bacteria in the respiratory microbiota. (**A**) Kaplan–Meier curves for the clinical variables with a statistical significant difference in the probability survival. Variables were classified into two categories specified in the legend “factor”. All curves were constructed with hospitalization days and outcome (either deceased or alive). (**B**) Differentially abundant microbes associated with patients with the different risk factors for the clinical variables obtained through Kaplan–Meier curves. Risk factor (either high or low) corresponds to the result of the survival curves (panel A). *ASV* Amplicon Sequence Variant, *APACHE* Acute Physiology and Chronic Health Evaluation, *BUN* Blood Urea Nitrogen. Color code corresponds to the LDA score obtained from LefSe analysis. Only features with a LDA score higher than 1.5 and a *p* < 0.01 were included.

The Kaplan–Meier survival curves allowed us to detect clinical variables that correlated significantly with the probability of survival (Fig. 4A). In particular, we found that APACHE scores above eight points (Kaplan–Meier curves, *p* = 0.05), Blood Urea Nitrogen (BUN) levels below 40 mg/dl (Kaplan–Meier curves, *p* = 0.01), lymphocytes below 1.25 × 10^3^/µl (Kaplan–Meier curves, *p* = 0.008), myoglobin above 110 ng/ml (Kaplan–Meier curves, *p* = 0.05), troponin above 3.5 ng/ml (Kaplan–Meier curves, *p* = 0.01), and urea above 80 mg/dl (Kaplan–Meier curves, *p* = 0.03), represent a high risk by negatively affecting the probability of survival.

The Lefse analysis allowed us to detect enriched or depleted bacteria in the different risk factor groups for the variables analyzed (Fig. 4B). We found depleted *Neisseria subflava* in the high-risk samples for troponin and APACHE. On the other hand, *Veillonella dispar* was found interestingly depleted in the low-risk samples for APACHE, BUN, myoglobin, and urea. However, we also found some bacterial groups to be consistently enriched in the high-risk samples for several clinical variables. For example, *Corynebacterium* was found to be enriched in the high-risk samples for lymphocytes count and urea, while *Actinomyces* was enriched for BUN and urea. Additionally, four ASV´s of the genus *Prevotella* (*Prevotella melaninogenica*; *Prevotella*; [*Prevotella*]; [*Prevotella*]_s) were found significantly enriched in the high-risk samples for myoglobin, BUN, troponin, and lymphocyte count.

### The structure of the respiratory microbiota is different between patients with different severity levels of COVID-19

**Figure 5:**
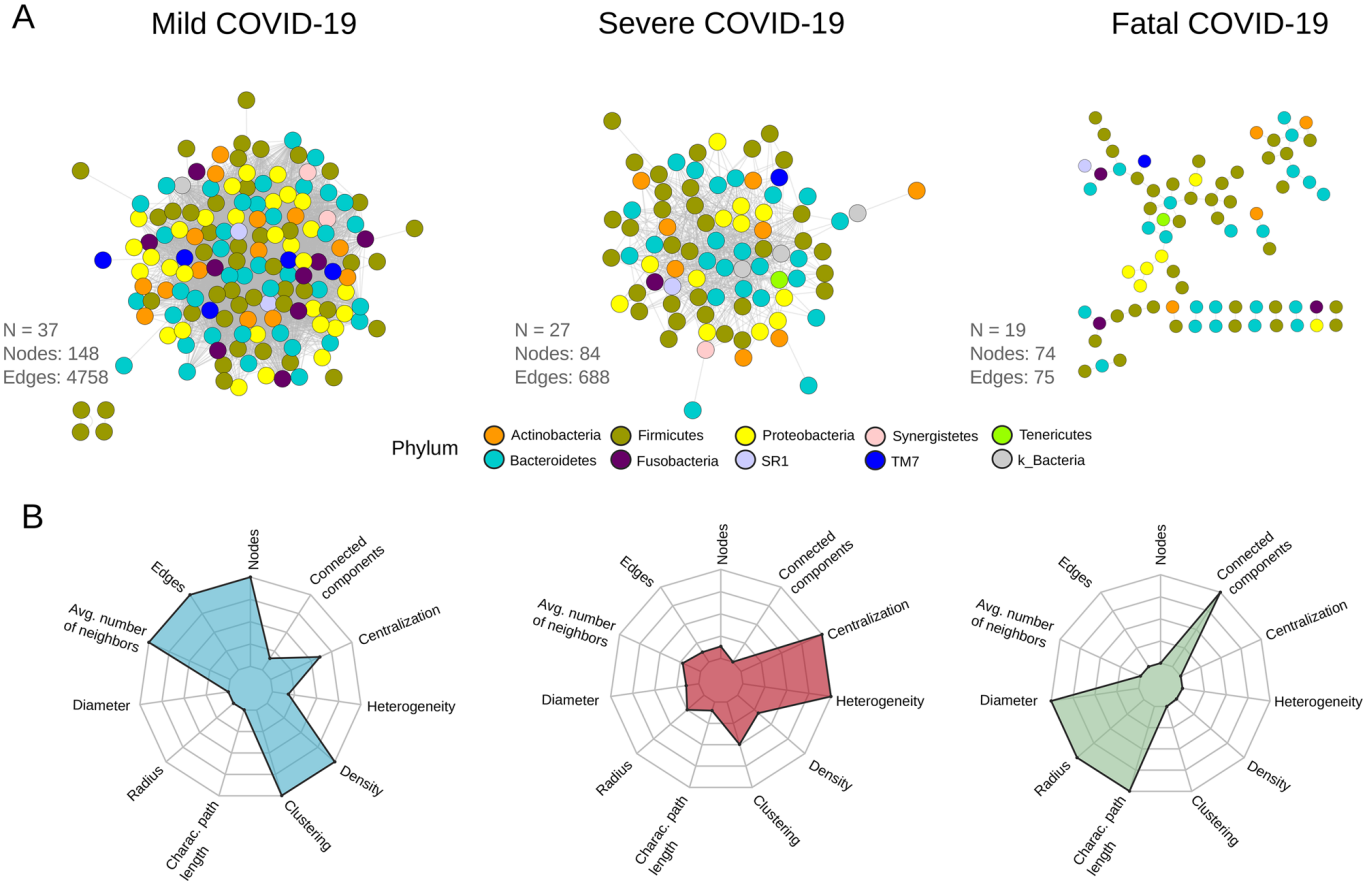
Network structure of the respiratory microbiota between patients with different severity levels of COVID-19. (**A**) Co-occurrence/exclusion networks for patients with mild, severe and fatal COVID-19. Each node represents a microbial group at ASV level and each edge an interaction (either co-occurrence or co-exclusion). Colors denote phylum identity. Number of samples used to construct the network (N), number of nodes, and number of edges are reported in the figure. (**B**) Spider chart of the topological metrics associated to each network.

Network analysis for the microbiota associated with the severity levels for COVID-19 revealed differences at the structural level (Fig. 5A,B). Graphical representation of the networks showed a different arrangement for each and a continuum of loss of complexity across COVID-19 severity groups (from mild to fatal) (Fig. 3A). In particular, the microbiota network associated with mild COVID-19 was the largest and most connected (nodes = 148, edges = 4758) compared to severe COVID-19 (nodes = 84, edges = 688) and fatal COVID-19 (nodes = 74, edges = 75). Interestingly, in patients with fatal outcomes, the respiratory microbiota network was highly disaggregated and poorly connected with multiple isolated nodes (nodes = 74, edges = 75).

The metric calculation of the networks illustrates that the topology associated with each COVID-19 severity level is different (Fig. 3B, Supplementary Table S1). For example, the mild COVID-19 network showed the highest values for the average number of neighbors, density, and clustering. By contrast, the severe disease network was characterized by higher centralization and heterogeneity, whereas the fatal disease network showed the highest values for diameter, characteristic path length, and connected components.

## Discussion

Due to the massive efforts of the global research community, many investigations on the role of the microbiota in COVID-19 have appeared. Despite the clear focus on gut microbiota^6,21,22^ there have been some studies that have characterized the respiratory microbiota and its impact on COVID-19. However, most of them focus on comparing patients with COVID-19 to those without COVID-19^13–20^, and do not include disease severity levels thus hindering information that could potentially help to understand disease progression. In turn, to date, few studies have characterized the respiratory microbiota in COVID-19 among severity levels^23,24^. Here, we analyzed the respiratory microbiota from an eco-evolutionary perspective, by testing microbial composition and community structure of a large cohort of patients with different levels of severity (mild, severe, and fatal) and linked the results to clinical variables to gain insight into the mechanisms by which the microbiota may affect host response against the disease.

As in recent studies investigating the etiology of COVID-19^3,5^, we found that demographic and health-related factors showed strong associations with severity. Male sex, high values for BMI, age over 50 years, and previous antibiotic treatment were significantly associated with patients with fatal COVID-19 (Table 1), which potentially favor the development of a fatal state of the disease.

Similarly, in previous work exploring the respiratory microbiota associated with COVID-19^24–26^, we found significantly lower microbial diversity in the microbiota of COVID-19 patients than in the healthy controls (Fig. 1A). This result is relevant since, in general, a higher diversity correlates with a better response of microbial systems against perturbations (e.g., disease). A more diverse microbiota may persist after disease (e.g., by functional redundancy) or may recover to a previous state (e.g., resilience)^27^, having direct consequences on host health^11^.

Furthermore, we found differences in the abundance of some bacteria between our study groups (Fig. 1A). In particular, as in other respiratory diseases^28^, we observed a higher ratio of Firmicutes/Bacteroidetes in patients with COVID-19. Firmicutes was detected highly abundant while Bacteroidetes was especially decreased in the microbiota associated with COVID-19 patients. This is of particular interest since, in murine models, it has been shown that Bacteroidetes can down-regulate the ACE2 expression^29^. Although the correlation was observed in the gut microbiota, the low abundance of members of that phylum in the severe and fatal patients in this study opens the question of whether this process may also take place in the respiratory tract.

Regarding the composition at the genus level, most of the differences we found were in potentially pathogenic bacteria (Fig. 1B). A gradual increase of *Streptococcus* was identified from mild to fatal COVID-19 cases. Although *Streptococcus* is usually a commensal member of the respiratory microbiota, it can become pathogenic in the face of environmental disturbances. In higher abundance, this genus has been associated with viral acute respiratory infections^30,31^. In addition, genera such as *Veillonella*, *Staphylococcus*, and *Actinomyces* also showed high abundances at different COVID-19 severity levels. Specifically, *Veillonella* and *Actinomyces* have been found as opportunistic pathogens in COVID-19^32^. Additionally, *Staphylococcus* is one of the most common causative agents of secondary infections in respiratory diseases such as influenza^33^.

Regarding the characterization of the microbiota in the lower respiratory tract, we detected opposite patterns in the abundances of some phyla and genera between severe and fatal COVID-19 (Supplementary Fig. S1), suggesting that the phenomena that we observe in the upper tract, due to particular environmental conditions could be different in the lungs. For instance, respiratory diseases generally entail inflammatory processes that increase mucus production which in turn, favor the presence of biofilms and anaerobic bacteria in the lungs^34^. This could be the reason behind the depletion of aerobic bacteria such as *Staphylococcus* and the increase of anaerobic groups such as *Streptococcus*,* Abiotrophia*, and *Mycoplasma* in the samples of the lower respiratory tract.

In terms of beta diversity, we found some differences between the analyzed groups. For example, we observed in the PCoA analysis that the samples belonging to severe and fatal COVID-19, as well as to the non-COVID-19-pneumonia group, are more similar in terms of microbial composition, than those from healthy controls and mild COVID-19 patients (Fig. 2A). Moreover, the PCoA analysis displayed a change in the tendency of variation between some of the analyzed groups. This observation implies that the features that define the microbial variation within each group are distinct, at least between mild COVID-19/healthy controls and severe forms of the disease, including non-COVID-19 pneumonia. Furthermore, our dysbiosis analysis let us detect that COVID-19 microbiota showed higher levels of dysbiosis than from the healthy controls (Supplementary Fig. S2). Several MADs exhibit this behavior in which microbiota instability (dysbiosis) is present not by the dominance of one or a few bacteria but because of a higher heterogeneity/stochasticity of microbial groups^12^. Dysbiosis involves a disruption in the bidirectional interactions between the host immune system and the microbial communities, which can alter the functions provided by these communities and reshape the entire host-microbiota interaction^11,35^. Microbiota stability has been shown to be a hallmark of host health and homeostasis^9,11,31^. For example, some reports suggest that there is a homeostatic mechanism that maintains the lung epithelium in a state of interferon primacy with antiviral activity against other respiratory infections such as influenza. This particular antiviral response is stimulated by specific pathogen-associated molecular patterns (PAMPs) that are induced by microbial communities^36^. Furthermore, in other viral diseases it has been shown that, due to different mechanisms (e.g. alteration of the epithelium and increased adhesion of respiratory pathogens), microbial dysbiosis can promote viral infection, potentially including SAR-CoV-2^24^.

A common question when studying MADs is whether dysbiosis favors the disease or is caused by it. In the case of COVID-19, the clinical outcome is highly correlated with comorbidities such as hypertension, diabetes, and obesity^10^, which are often associated with dysbiosis in the gut microbiota^32^. This observation, together with the highly distributed antibiotic consumption in COVID-19 patients (53.6% in our cohort, regardless of severity), warrants a reflection on the possibility that most of the patients could be dysbiotic at the time of illness. This particular scenario in which a previous dysbiotic microbiota caused by comorbidities and/or antibiotic consumption may affect susceptibility and outcome of COVID-19 has been suggested previously^20,37^. In other respiratory diseases such as COPD and asthma, it has been shown that dysbiosis in the respiratory microbiota can lead to a dysregulated immune response, increasing inflammatory processes^9,11,38^. Considering that aberrant immune responses are determinant in the progression of COVID-19, a previous dysbiotic respiratory microbiota could be affecting disease progression.

LefSe analysis (Fig. 3B) showed a differential abundance of microbial groups. For example, we found that most of the groups associated with the healthy controls belong to the so-called "normal" respiratory microbiota (e.g., *Streptococcus, Oribacterium,* and *Veillonellaceae*)^39^. By contrast, when we looked at the results of the microbiota associated with the COVID-19 and non-COVID-19 pneumonia groups, other potentially pathogenic microbial groups appeared. Specifically, in patients with non-COVID-19 pneumonia, we found bacteria associated with nosocomial infections such as *Corynebacterium*^40,41^. For mild COVID-19, we found some microbial groups associated with disease or bacteremia like *Prevotella melaninogenica, V. parvula* and *Neisseria subflava*^10,41^. For the case of severe COVID-19, we found *Megasphaera* which has been associated with the risk of ventilator-associated pneumonia (VAP) in other studies characterizing the microbiota of COVID-19^16^. Additionally, we found *Rothia dentocariosa* very abundant in deceased patients. This bacteria has been found as a causative agent of secondary pneumonia in H1N1 infection^33^ and more recently has been associated with disease progression in previous studies characterizing the respiratory microbiota of COVID-19, proposing it as a biomarker for the disease^26,42^.

From a clinical standpoint, it makes sense that a higher mortality predictor such as APACHE score correlates with poor survival in patients with COVID-19. Other clinical factors such as BUN or urea have also been used as markers of severity in respiratory diseases such as community-acquired pneumonia^43^. Acknowledging that there are multiple pathophysiological considerations still unexplained in SARS-CoV-2 infection, and the multisystemic involvement that has been observed in COVID-19^44^, biochemical markers of organ dysfunction such as lymphopenia, elevated myoglobin, and serum troponin levels, such as those found in this study, can help predict mortality in these patients^45^. Although the association of these factors with specific microbial groups in the respiratory tract has not been previously reported, the findings of this work pave the way for further study of the relationship between respiratory microbiota and clinical outcomes. The identification of pathogenic bacteria such as *Actinomyces, Prevotella,* and *Corynebacteriu*m in association with two or more clinical factors further supports the current line of research attempting to correlate the gut-lung axis with pulmonary disease^46^.

Recent studies, as well as this work, suggest that particularly anaerobic bacteria inhabiting the respiratory tract may be involved in the pathogenesis of COVID-19 and the host immune system. For example, in patients with high levels of blood urea, we found *Corynebacterium*, which has been previously linked to hyperuricemia^47^, and has also been found as an opportunistic pathogen in patients with immunosuppression or severe disease^48^. In addition, *Prevotella* has also been found to be increased in studies with patients with severe disease and has been linked to cardiac injury and high-risk mortality^45,49^. In this work, we found this specific genus associated with four clinical variables predictive of mortality in patients with COVID-19 (Fig. 4A,B). This finding is of particular interest considering previous evidence that *Prevotella* potentiates a Th17-mediated response through IL-8, CCL20, and IL-6 secretion^49,50^; both the Th17 response and its cytokines are currently associated with the host immune response to SARS-CoV-2^51^.

Finally, the co-occurrence arrangement of ecological networks lets us identify structural patterns that reflect variations in the biological properties of COVID-19- associated microbial communities. For example, we found that all networks are distinguishable in terms of topological metrics such as density, clustering, and heterogeneity. It is worth mentioning that such metrics are potentially related to the stability of the systems as well as to other ecological properties such as resilience and redundancy^52^. In particular, we found a striking pattern of reduction in structural complexity from mild to fatal COVID-19. The loss of complexity manifests itself in a reduction in the number of nodes, edges (connections), density, and clustering, and moves from a highly connected and dense network (mild COVID-19) to a highly disaggregated and disconnected network (fatal COVID-19) (Fig. 5A,B, Supplementary Table S1).

These structural changes can lead to the generation of hypotheses about the consequences at the microbial community level. For example, changes in structural patterns could potentially be reflected in alterations in the ecological relationships between microorganisms. A common feature in MADs is that commensal/neutral bacteria can become pathogenic in the face of disease^32^. That is the case of bacteria such as *Prevotella*, *Veillonella*, *Streptococcus*, *Actinomyces*, or *Megasphaera*, which have been found as opportunistic pathogens in other COVID-19 microbiota characterization studies^16,30–32^ and were also found in this study [severe and fatal associated microbiota (Fig. 3B)]. The shift from neutral to deleterious interactions in specific bacteria could be the result of a loss of interactions that maintain the function and stability of microbial systems. This, in turn, could lead to exacerbated growth of potentially pathogenic microbial groups, but also the depletion of beneficial bacteria, altering the entire environment and possibly compromising the functions provided by the microbiota to the host.

## Conclusions

Overall, this work provides insight into the role of the respiratory microbiota in COVID-19 disease. Although experimental validation is needed, our data suggest that environmental and host-related factors could be affecting the respiratory microbiota prior to SARS-CoV-2 infection, potentially compromising the immunological response of the host against disease and promoting secondary bacterial infections. We hypothesize that high levels of dysbiosis and poor microbial structural complexity in the respiratory microbiota of COVID-19 patients could be the result of antibiotic intake and comorbidities. Although further validation of our results is needed, an earlier dysbiotic state as that found in our study may have consequences at the host and microbial community level. On the one hand, increased dysbiosis in diseased patients could be modifying the PAMPs that stimulate a homeostatic antiviral response, allowing better conditions for SAR-CoV-2 replication. Additionally, the loss of structural complexity may lead to the emergence of opportunistic pathogens that, through ecological competition, may cause depletion of beneficial bacteria and promote secondary bacterial infections that worsen the clinical outcome. In summary, the findings of this work contribute to understanding the pathology of COVID-19 by identifying the respiratory microbiota as a potential factor affecting disease outcomes. It is worth mentioning that the main limitation of our study is the number of healthy subjects enrolled, which were all the eligible individuals in our research center at that time. Further research involving a larger number of healthy subjects is needed to confirm these findings. In addition, studies looking for the specific mechanisms by which dysbiotic respiratory tract microbiota compromise immune responses against virus infections are needed. Finally, further investigations using lower respiratory samples are needed to disentangle the behaviour of microbial communities in the lungs of patients with COVID-19.

## Methods

### Ethics statement

The Science, Biosecurity, and Bioethics Committee of the Instituto Nacional de Enfermedades Respiratorias revised and approved the protocol (B-1020). Informed consent was obtained from all subjects or their legal guardians (next of kin). Additionally, the Institution requested informed consent for the recovery, storage, and use of the biological remnant to research purposes. All experiments were performed following relevant guidelines and regulations.

### Study design

As part of a surveillance program at the Instituto Nacional de Enfermedades Respiratorias Ismael Cosío Villegas (INER), 115 initial respiratory samples (oropharyngeal swabs, nasopharyngeal swabs, and tracheal aspirates) were collected between March 2020 and October 2020. Additionally, we included seven subjects without respiratory symptoms and negative SARS-CoV-2 RT-PCR test (healthy), and five patients with pneumonia that were hospitalized but negative to SARS-CoV-2 (non-COVID-19-pneumonia control group). Diagnostics for SARS-CoV-2 was performed with RT-qPCR according to the Berlin protocol, by using assays for the E, RdRP, and N genes (LightMix Modular SARS-CoV-2, TibMOLBIOL, Berlin, Germany) following the method reported in Corman et al.^53^ recommended by the WHO. Patients with COVID-19 were classified into three mutually exclusive categories of severity: (a) mild COVID-19 (patients with moderate symptoms who did not require hospitalization), (b) severe COVID-19 (patients who required hospitalization and were subject to Invasive Mechanical Ventilation (IMV)), and (c) fatal COVID-19 (deceased patients). Overall, a total of 37 patients with mild disease, 38 with severe disease, and 40 with fatal outcomes were included in the study.

### DNA extraction and 16S rRNA sequencing

Respiratory samples for all 127 patients, either nasopharyngeal swabs, oropharyngeal swabs, or tracheal aspirates, were collected and centrifuged for 15 min at 4800×*g*, and the pellet was used for DNA extraction. DNA was extracted using the QIAmp Cador Pathogen Mini Kit extraction (Qiagen N.V., Hilden, Germany) according to the manufacturer´s instructions. V3–V4 16S rRNA region was amplified by PCR using the primers reported by Klindworth et al.^54^ (for more information see Supplementary Material S1). Library preparation was done according to the Illumina 16S metagenomic sequencing protocol with few modifications. Briefly, 16S amplicons were purified with the DNA clean & concentrator kit (Zymo Research, Irvine Cal., USA). Dual indices and Illumina sequencing adapters were attached in a second PCR step using Nextera XT Index Kit V2 (Illumina, San Diego Cal., USA). Finally, amplicons were purified, pooled in equimolar concentrations, and sequenced in a MiSeq Illumina instrument generating paired-end reads of 250 bp.

### Sequence data processing

Illumina raw sequences were processed with QIIME2 (v2020.8)^55^. Sequences denoising, quality filtering, and chimera checking were performed with DADA2 (v1.18)^56^. From the original number of reads (13,533,440), we retained a total of 9,499,204 with an average of 73,637 sequences per sample and a length of 225 bp. A total of 4514 Amplicon Sequence Variants (ASVs) were generated, aligned with MAFFT (v7)^57^, and used to construct a phylogeny with fasttree (v2.1.11)^58^. ASVs taxonomy was assigned with the Näive Bayes classifier sklearn (v0.23.1)^59^ using the Greengenes 13.8 database^60^. All ASVs identified as mitochondria (N = 10), and chloroplast (N = 32) were removed.

### Diversity, compositional, and statistical analyses

Since our sample set contains samples from the upper (Oropharyngeal swabs [OPS], Nasopharyngeal swabs [NPS]) and lower (Tracheal aspirates [TA]) respiratory tract, and these sites are known to differ in microbial composition, we used only upper respiratory samples for the main analyses. After this process we retained a total of 95 samples (mild COVID-19 = 37, severe COVID-19 = 27, fatal COVID-19 = 19, healthy control = 7, and non-COVID-19-pneumonia = 5). In addition, we also characterized TA samples to compare levels of severity in the microbiota of the lower respiratory tract.

All 16S analyses were performed using R v4.0.2 in RStudio v1.3.1 and the packages ggplot2 (v3.3.3)^61^, vegan (v2.5.7)^62^, microbiome (v2.1.28)^63^, phyloseq (v1.32.0)^64^, fmsb (v0.7.0)^65^, randomcolorR (v1.1.0.1)^66^, and CommEcol (v1.7.0)^67^.

#### Composition analyses

To determine if the respiratory microbiota of patients with different severity levels of COVID-19 and controls differed in microbial composition, we constructed stacked barplots of relative abundanceat phylum and genus levels. The relative abundance of each taxon was calculated with phyloseq and plotted with ggplot2 R packages. The colors were randomly assigned using randomcoloR R package.

#### Alpha diversity

We calculated the Shannon–Wiener diversity index with the "microbiome" R package. To detect potential differences between groups we conducted a Wilcoxon rank-sum test in the "vegan" R package.

#### Beta diversity

We carried out a Principal Coordinates Analysis (PCoA) with weighted UniFrac distance at ASV level in the "phyloseq" R package. Potential differences in beta diversity were addressed with a Permutational Analysis of Variance (PERMANOVA) with 999 permutations performed with the "vegan" R package. Additionally, we tested for dispersion and stochasticity as a proxy of dysbiosis in microbial communities^68^. For this purpose, we calculated the Ružička similarity metric in the "CommEcol" R package and performed a Wilcoxon rank-sum test to detect potential statistical differences between healthy controls and diseased groups in their intra-treatment sample similarities. Dysbiosis was assumed when the similarities between the healthy microbiota samples were significantly higher than the similarities between the diseased microbiota samples^12^.

Finally, to detect differentially abundant taxa associated with severity levels and controls, we performed a Linear Discriminant Analysis (LDA) with effect size (LefSe) at the ASV level using the web-based tool MicrobiomeAnalyst^69^. Only taxa with an LDA score higher than 1.5 and a *p*-value < 0.01 obtained from the Kruskal–Wallis test were used.

### Clinical data analyses

To analyze the clinical data associated with our patient’s cohort, we transform each clinical variable into a binomial category according to its data distribution. We used cut-points based on the 25 and 75 percentiles for each variable. For example, for a given variable, we classified all samples with values above or equal to the 75 percentile as "1", and all samples with values under the 75 percentile as "2". Subsequently, for all clinical variables (N = 65) we constructed Kaplan–Meier survival curves in SPSS Statistics (version 21) (Chicago, Illinois, USA) by using the hospitalization days as time variable, the mortality status (either deceased or alive) as a dependent variable, and the specific clinical qualitative variables as exposure variable. Only those curves statistically (*p* < 0.05) and biologically meaningful were retained for the subsequent analyses.

In addition, to determine if there were differentially abundant bacteria associated with the several risk factors for the clinical variables obtained from the Kaplan–Meier curves, we performed a second LefSe analysis. From this result, only taxa with an LDA score higher than 1.5 and a *p*-value < 0.01 according to the Kruskal–Wallis test were used.

### Network structure inference

We inferred the network structure for the microbiota associated with patients with different severity levels of COVID-19. Network calculation was performed in the software CoNet (v1.1.1 beta)^52^ by using read counts summarized at the ASV level. One network was constructed for each severity level (samples; mild COVID-19: 37, severe COVID-19: 27, and fatal COVID-19: 19). Only co-occurrences statistically supported by the three tested methods (Pearson, Spearman, and Kendall) with a correlation > 0.85 and a *p*-value < 0.01 were established as edges in the graphs. Also, we applied a multi-test correction using the Benjamini–Hochberg procedure. Network visualization was performed in Cytoscape (v. 3.8.2)^70^.

To further characterize the structure we computed metrics of the topology of each network using the NetworkAnalyzer plug-in in Cytoscape^70^ and visualized them with a spider chart constructed in fmsb R package.

## Supplementary Information

Supplementary Information.

## Data Availability

The raw data of 16S rRNA gene generated during the present study are available in the NCBI Sequence Read Archive (SRA) (https://www.ncbi.nlm.nih.gov/sra/) with the accession PRJNA726205.

## References

[1] WHO. Weekly epidemiological update on COVID-19-11 May 2021. *11 May* 32 (2021). https://www.who.int/publications/m/item/weekly-epidemiological-update-on-covid-19---11-may-2021. Accessed 12 May 2021.

[2] Baloch S, Baloch MA, Zheng T, Pei X (2020). The coronavirus disease 2019 (COVID-19) pandemic. Tohoku J. Exp. Med..

[3] Sharma, R., Agarwal, M., Gupta, M., Somendra, S. & Saxena, S. K. Clinical characteristics and differential clinical diagnosis of novel coronavirus disease 2019 (COVID-19). in *Coronavirus Disease 2019 (COVID-19): Epidemiology, Pathogenesis, Diagnosis, and Therapeutics* (ed. Saxena, S. K.) 55–70 (Springer, 2020). doi:10.1007/978-981-15-4814-7_6

[4] Guan W (2020). Comorbidity and its impact on 1590 patients with COVID-19 in China: A nationwide analysis. Eur. Respir. J..

[5] Pijls BG (2021). Demographic risk factors for COVID-19 infection, severity, ICU admission and death: A meta-analysis of 59 studies. BMJ Open.

[6] Yeoh YK (2021). Gut microbiota composition reflects disease severity and dysfunctional immune responses in patients with COVID-19. Gut.

[7] Man WH, De Steenhuijsen Piters WAA, Bogaert D (2017). The microbiota of the respiratory tract: gatekeeper to respiratory health. Nat. Rev. Microbiol..

[8] Chung H (2012). Gut immune maturation depends on colonization with a host-specific microbiota. Cell.

[9] Budden KF (2019). Functional effects of the microbiota in chronic respiratory disease. Lancet Respir. Med..

[10] Bao L (2020). Oral microbiome and SARS-CoV-2: Beware of lung co-infection. Front. Microbiol..

[11] Li KJ (2019). Dysbiosis of lower respiratory tract microbiome are associated with inflammation and microbial function variety. Respir. Res..

[12] Ma Z (2020). Testing the Anna Karenina Principle in human microbiome-associated diseases. Science.

[13] Rueca M (2021). Investigation of nasal/oropharyngeal microbial community of covid-19 patients by 16s rdna sequencing. Int. J. Environ. Res. Public Health.

[14] Iebba V (2021). Profiling of oral microbiota and cytokines in COVID-19 patients. Front. Microbiol..

[15] Engen PA (2021). Nasopharyngeal microbiota in SARS-CoV-2 positive and negative patients. Biol. Proced. Online.

[16] Lloréns-Rico, V. *et al.* Mechanical ventilation affects respiratory microbiome of COVI-19 patients and its interactions with the host. *medRxiv* (2021).

[17] Xu R (2021). Progressive deterioration of the upper respiratory tract and the gut microbiomes in children during the early infection stages of COVID-19. J. Genet. Genomics.

[18] Rosas-Salazar, C. *et al.* SARS-CoV-2 infection and viral load are associated with the upper respiratory tract microbiome. *J. Allergy Clin. Immunol.* (2021). 10.1016/j.jaci.2021.02.001 PMC7871823 33577896

[19] Ma S (2021). Metagenomic analysis reveals oropharyngeal microbiota alterations in patients with COVID-19. Signal Transduct. Target. Ther..

[20] Wu Y (2021). Altered oral and gut microbiota and its association with SARS-CoV-2 viral load in COVID-19 patients during hospitalization. NPJ Biofilms Microbiomes.

[21] Gu S (2020). Alterations of the gut microbiota in patients with coronavirus disease 2019 or H1N1 influenza. Clin. Infect. Dis..

[22] Zuo T (2020). Alterations in gut microbiota of patients with COVID-19 during time of hospitalization. Gastroenterology.

[23] Zhong H (2021). Characterization of respiratory microbial dysbiosis in hospitalized COVID-19 patients. Cell Discov..

[24] Soffritti I (2021). Oral microbiome dysbiosis is associated with symptoms severity and local immune/inflammatory response in COVID-19 patients: A cross-sectional study. Front. Microbiol..

[25] Zhang, H. *et al.* Metatranscriptomic characterization of COVID-19 identified a host transcriptional classifier associated with immnune signaling. *Clin. Infect. Dis.* (2020). 10.1093/cid/ciaa663 PMC7314197 32463434

[26] Xu R (2021). Temporal association between human upper respiratory and gut bacterial microbiomes during the course of COVID-19 in adults. Commun. Biol..

[27] Allison SD, Martiny JBH (2009). Resistance, resilience, and redundancy in microbial communities. Light Evol..

[28] Wang B, Yao M, Lv L, Ling Z, Li L (2017). The human microbiota in health and disease. Engineering.

[29] Geva-Zatorsky N (2017). Mining the human gut microbiota for immunomodulatory organisms. Cell.

[30] Teo SM (2015). The infant nasopharyngeal microbiome impacts severity of lower respiratory infection and risk of asthma development. Cell Host Microbe.

[31] Lynch SV (2016). The lung microbiome and airway disease. Ann. Am. Thorac. Soc..

[32] Ferreira C, Viana SD, Reis F (2021). Is gut microbiota dysbiosis a predictor of increased susceptibility to poor outcome of COVID-19 patients? An update. Microorganisms.

[33] Prakash R, Sangeetha S, Lakshminarayana SA, Sunil Kumar DC (2016). Secondary pneumonia due to *Rothia mucilaginosa* in H1N1 patient. J. Int. Med. Dent..

[34] Wypych TP, Wickramasinghe LC, Marsland BJ (2019). The influence of the microbiome on respiratory health. Nat. Immunol..

[35] Kumar P, Chander B (2020). COVID 19 mortality: Probable role of microbiome to explain disparity. Med. Hypotheses.

[36] Bradley KC (2019). Microbiota-driven tonic interferon signals in lung stromal cells protect from influenza virus infection. Cell Rep..

[37] Kalantar-Zadeh K, Ward SA, Kalantar-Zadeh K, El-Omar EM (2020). Considering the effects of microbiome and diet on SARS-CoV-2 infection: Nanotechnology roles. ACS Nano.

[38] Khatiwada S, Subedi A (2020). Lung microbiome and coronavirus disease 2019 (COVID-19): Possible link and implications. Hum. Microbiome J..

[39] Huffnagle GB, Dickson RP, Luckacs NW (2017). The respiratory tract microbiome and lung inflammation: A two-way street. Mucosal Immnunol..

[40] Renom F (2014). Respiratory infection by *Corynebacterium striatum*: Epidemiological and clinical determinants. New Microbes New Infect..

[41] Zimmermann A (2017). Atopobium and fusobacterium as novel candidates for sarcoidosis-associated microbiota. Eur. Respir. J..

[42] Marotz C (2021). SARS-CoV-2 detection status associates with bacterial community composition in patients and the hospital environment. Microbiome.

[43] Lim WS (2009). British Thoracic Society guidelines for the management of community acquired pneumonia in adults: Update 2009. Thorax.

[44] Bohn MK (2020). Pathophysiology of COVID-19: Mechanisms underlying disease severity and progression. Physiology.

[45] Zheng YY, Ma YT, Zhang JY, Xie X (2020). COVID-19 and the cardiovascular system. Nat. Rev. Cardiol..

[46] Fromentin M, Ricard JD, Roux D (2021). Respiratory microbiome in mechanically ventilated patients: A narrative review. Intensive Care Med..

[47] Tauch A, Fernández-Natal I, Soriano F (2016). A microbiological and clinical review on *Corynebacterium kroppenstedtii*. Int. J. Infect. Dis..

[48] Pan L (2020). Abnormal metabolism of gut microbiota reveals the possible molecular mechanism of nephropathy induced by hyperuricemia. Acta Pharm. Sin. B.

[49] Chakraborty S (2020). Metagenome of SARS-Cov2 patients in Shenzhen with travel to Wuhan shows a wide range of species: Lautropia, Cutibacterium, Haemophilus being most abundant and Campylobacter explaining diarrhea. OSF Prepr..

[50] Larsen JM (2017). The immune response to *Prevotella* bacteria in chronic inflammatory disease. Immunology.

[51] De Biasi S (2020). Marked T cell activation, senescence, exhaustion and skewing towards TH17 in patients with COVID-19 pneumonia. Nat. Commun..

[52] Faust K, Raes J (2016). CoNet app: Inference of biological association networks using Cytoscape. F1000 Res..

[53] Corman VM (2020). Detection of 2019 novel coronavirus (2019-nCoV) by real-time RT-PCR. Eurosurveillance.

[54] Klindworth A (2013). Evaluation of general 16S ribosomal RNA gene PCR primers for classical and next-generation sequencing-based diversity studies. Nucleic Acids Res..

[55] Bolyen E (2019). Reproducible, interactive, scalable and extensible microbiome data science using QIIME 2. Nat. Biotechnol..

[56] Callahan BJ (2016). DADA2: High resolution sample inference from Illumina amplicon data. Nat. Methods.

[57] Katoh K, Misawa K, Kuma KI, Miyata T (2002). MAFFT: A novel method for rapid multiple sequence alignment based on fast Fourier transform. Nucleic Acids Res..

[58] Price MN, Dehal PS, Arkin AP (2010). FastTree 2: Approximately maximum-likelihood trees for large alignments. PLoS ONE.

[59] Bokulich NA (2018). Optimizing taxonomic classification of marker-gene amplicon sequences with QIIME 2’s q2-feature-classifier plugin. Microbiome.

[60] McDonald D (2012). An improved Greengenes taxonomy with explicit ranks for ecological and evolutionary analyses of bacteria and archaea. ISME J..

[61] Wickham H (2016). ggplot2: Elegant Graphics for Data Analysis.

[62] Oksanen J (2013). Community Ecology Package.

[63] Lahti L, Shetty S (2017). Tools for microbiome analysis in R.

[64] McMurdie PJ, Holmes S (2013). Phyloseq: An R package for reproducible interactive analysis and graphics of microbiome census data. PLoS ONE.

[65] Nakazawa M (2019). fmsb: Functions for Medical Statistics Book with Some Demographic Data.

[66] Ammar R (2019). randomcoloR: Generate Attractive Random Colors.

[67] Melo AS (2016). CommEcol: Community Ecology Analyses.

[68] Ning D, Deng Y, Tiedje JM, Zhou J (2019). A general framework for quantitatively assessing ecological stochasticity. Proc. Natl. Acad. Sci. USA.

[69] Dhariwal A (2017). MicrobiomeAnalyst: A web-based tool for comprehensive statistical, visual and meta-analysis of microbiome data. Nucleic Acids Res..

[70] Shannon P (2003). Cytoscape: A Software Environment for Integrated Models. Genome Res..

