# Supplementary material for: Dysbiosis and structural disruption of the respiratory microbiota in COVID-19 patients with severe and fatal outcomes
Source: Sci Rep. 2021 Oct 29;11:21297. doi: 10.1038/s41598-021-00851-0 (PMC8556282; doi:10.1038/s41598-021-00851-0)
Supplement: Supplementary file 1 — Supplementary Information. [file 41598_2021_851_MOESM1_ESM.docx]

**SUPPLEMENTARY MATERIAL**

Dysbiosis and structural disruption of the respiratory microbiota in COVID-19 patients with severe and fatal outcomes

Alejandra Hernández-Terán, Fidencio Mejía-Nepomuceno, María Teresa Herrera, Omar Barreto, Emma García, Manuel Castillejos, Celia Boukadida, Margarita Matias-Florentino, Alma Rincón-Rubio, Santiago Avila-Rios, Mario Mújica-Sánchez, Ricardo Serna-Muñoz, Eduardo Becerril-Vargas, Cristobal Guadarrama-Pérez, Víctor Hugo Ahumada-Topete, Sebastián Rodríguez, José Arturo Martínez-Orozco, Jorge Salas-Hernández, Rogelio Pérez-Padilla, Joel Armando Vázquez-Pérez.

**S1. Specifics for 16S rRNA gene amplification**

**PCR cycles:**

95°C for 3 min, followed by 35 cycles at 95° C for 30s, 55°C for 30s, and 72°C for 30s, with a final extension at 72°C for 5 min

**Primers used:**

F - 5´ CCTACGGGNGGCWGCAG 3´

R - 5´GACTACHVGGGTATCTAATCC 3´

**Figure S1. Diversity and composition of the microbiota in the lower respiratory tract of patients with severe and fatal COVID-19. A:** Stacked barplot illustrating the microbial composition between patients with severe and fatal COVID-19 at phylum level. **B:** Stacked barplot illustrating the microbial composition between patients with severe and fatal COVID-19 at genus level. **C:** Shannon diversity index among COVID-19 severity levels in the lower respiratory tract. **D:** Differentially abundant taxa obtained through LefSe analysis for each group. Only features with a LDA score higher than 1.5 (or -1.5) and a *p* < 0.01 were included. **ASV:** Amplicon Sequence Variant**. E:** Principal Coordinates Analysis (PCoA) with weighted Unifrac distance and PERMANOVA result that test differences in the community arrange among groups. Each color represents a group specified in the legend.

A

B

| **Comparison** | | **Mean similarity** | |  |
| --- | --- | --- | --- | --- |
| **Healthy** | **Diseased** | **Healthy** | **Diseased** | **Wilcoxon Test *p* value** |
| Healthy control | Mild COVID-19 | 0.94 | 0.89 | 0.05* |
|  | Severe COVID-19 | 0.94 | 0.88 | 0.01* |
|  | Fatal COVID-19 | 0.94 | 0.89 | 0.05* |
|  | Non-COVID-19-pneumonia | 0.94 | 0.97 | 0.08 |

**Figure S2.** **Ružička metric as a proxy of dysbiosis of the respiratory tract between patients with different severity levels for COVID-19 and controls. A:** Dotplot illustrating the intra-treatment similarity in terms of the Ružička metric. **B:** Table with the median for each treatment and the exact *p* value obtained in the pairwise comparisons. Dysbiosis was assumed when the similarities between the healthy microbiota samples were significantly higher than the similarities between the diseased microbiota samples (more dispersed).

**Table S1. Topological metrics for all the calculated co-occurrence networks.**

| **Metric** | **Mild COVID-19** | **Severe COVID-19** | **Fatal COVID-19** |
| --- | --- | --- | --- |
| Number of nodes | 148 | 84 | 74 |
| Number of edges | 4758 | 688 | 75 |
| Average number of neighbors | 65.86 | 16.35 | 2.31 |
| Diameter | 4 | 5 | 11 |
| Radius | 2 | 3 | 6 |
| Characteristic path length | 1.59 | 1.94 | 4.99 |
| Clustering | 0.788 | 0.47 | 0.155 |
| Density | 0.461 | 0.197 | 0.075 |
| Heterogeneity | 0.574 | 0.856 | 0.512 |
| Centralization | 0.391 | 0.563 | 0.127 |
| Connected components | 3 | 1 | 14 |
